# Streptolysin O concentration and activity is central to in vivo phenotype and disease outcome in Group A Streptococcus infection

**DOI:** 10.1038/s41598-021-97866-4

**Published:** 2021-09-24

**Authors:** Jenny Clarke, Murielle Baltazar, Mansoor Alsahag, Stavros Panagiotou, Marion Pouget, William A. Paxton, Georgios Pollakis, Dean Everett, Neil French, Aras Kadioglu

**Affiliations:** 1 Department of Clinical Immunology, Microbiology and Immunology, Institute of Infection and Global Health, University of Liverpool, Liverpool, UK; 2 Malawi-Liverpool-Wellcome Trust Clinical Research Programme, Queen Elizabeth Central Hospital, Blantyre, Malawi; 3 Faculty of Applied Medical Sciences, University of Al Baha, Al Baha, Saudi Arabia

**Keywords:** Microbiology, Bacteriology, Clinical microbiology, Pathogens

## Abstract

Group A Streptoccocus (GAS) is among the most diverse of all human pathogens, responsible for a range of clinical manifestations, from mild superficial infections such as pharyngitis to serious invasive infections such as necrotising fasciitis and sepsis. The drivers of these different disease phenotypes are not known. The GAS cholesterol-dependent cytolysin, Streptolysin O (SLO), has well established cell and tissue destructive activity. We investigated the role of SLO in determining disease outcome in vivo, by using two different clinical lineages; the recently emerged hypervirulent outbreak *emm* type 32.2 strains, which result in sepsis, and the *emm* type 1.0 strains which cause septic arthritis. Using clinically relevant in vivo mouse models of sepsis and a novel septic arthritis model, we found that the amount and activity of SLO was vital in determining the course of infection. The *emm* type 32.2 strain produced large quantities of highly haemolytic SLO that resulted in rapid development of sepsis. By contrast, the reduced concentration and lower haemolytic activity of *emm* type 1.0 SLO led to translocation of bacteria from blood to joints. Importantly, sepsis associated strains that were attenuated by deletion or inhibition of SLO, then also translocated to the joint, confirming the key role of SLO in determining infection niche. Our findings demonstrate that SLO is key to in vivo phenotype and disease outcome. Careful consideration should be given to novel therapy or vaccination strategies that target SLO. Whilst neutralising SLO activity may reduce severe invasive disease, it has the potential to promote chronic inflammatory conditions such as septic arthritis.

## Introduction

Group A Streptococcus (GAS), also called *Streptococcus pyogenes*, is a commensal of the human upper respiratory tract and also an important human pathogen, accounting for over 750 million infections every year^1,2^. GAS is able to produce a variety of pyogenic infections that range in severity and prevalence^3–5^. Diseases include pharyngitis, impetigo, cellulitis and life threatening infections such as streptococcal toxic shock syndrome, necrotising fasciitis, and sepsis^3,6^. The mechanisms that allow GAS to cause such diversity of disease types are unknown, however a number of studies have shown that a combination of bacterial and host-specific components may be involved^7^.

GAS strains can be typed based on the M-protein encoding *emm* gene sequence, of which there are over 200 known *emm* types^8^. The epidemiology of GAS infections has been changing globally over the last decade, with the emergence of new *emm* types and localised outbreaks a main feature^9^. Within *emm* types of GAS, isolates may be causative of a range of clinical outcomes, such that most lineages carry the potential for expression of a range of phenotypes that may determine the course and nature of infection. Previous studies have shown correlation between the host niche of recovered GAS clinical isolates and high concentrations of secreted virulence factors such as streptococcal pyrogenic exotoxin A, B, and C (SpeA, SpeB, and SpeC) and the haemolytic exotoxin streptolysin O (SLO)^10–12^. GAS phenotypic heterogeneity has further been linked to distinct clinical phenotypes by studies observing changes in virulence factor production, such as in streptokinase and capsular polysaccharide secretion, after GAS is passaged either ex vivo or in vivo^13–17^.

SLO is a haemolytic exotoxin belonging to the family of cholesterol dependent cytotoxins that also includes perfringolysin, pneumolysin, and listeriolysin^18–20^. SLO is toxic to diverse eukaryotic cell types including macrophages, neutrophils, and erythrocytes, as it targets cell membranes to form pores by interacting with cholesterol, that can lead to complete cell lysis^5,21–23^. SLO has a number of other biological effects on the host that act at different stages of infection, including hyper-stimulation and cell-meditated apoptosis of host immune cells such as neutrophils^11,24^. SLO is conserved in most GAS isolates, while differenetial expression has been linked to variation in cytotoxicity within and between *emm* types^25^. Early studies with SLO demonstrated that the purified toxin was lethal to mice and rabbits when injected intravenously, mainly due to cardiotoxicity^26,27^. More recently, there have been studies to assess the effects of biologically relevant concentrations of SLO in in vivo models. Limbago et al*.,* found that SLO-deficient GAS resulted in attenuated skin infections and Zhu et al*.,* reported a reduction in virulence in SLO-deficient GAS in an invasive wound infection model^28,29^.

Substantial gaps still exist however, in our understanding of the contributory role of the variation and amount of SLO production to overall GAS pathogenesis. In order to address this, we developed a specifically tailored SLO-ELISA to compare the production of SLO between a recently emerged hypervirulent outbreak strain (which resulted in an epidemic in Liverpool, UK, between 2010 and 2012) characterised as *emm* type 32.2 and invasive *emm* type 1.0. isolates^30^. Using in vivo GAS bacteraemia and novel septic arthritis models, we further investigated the role of SLO in establishing and maintaining different clinical phenotypes in vivo. In addition, we investigated the specific role of SLO in vivo*,* using a SLO deficient mutant strain in the background of an invasive outbreak *emm* type 32.2 isolate.

## Results

### In vivo characterisation of emm type 32.2 and 1.0 isolates in models of invasive GAS infection

**Figure 1:**
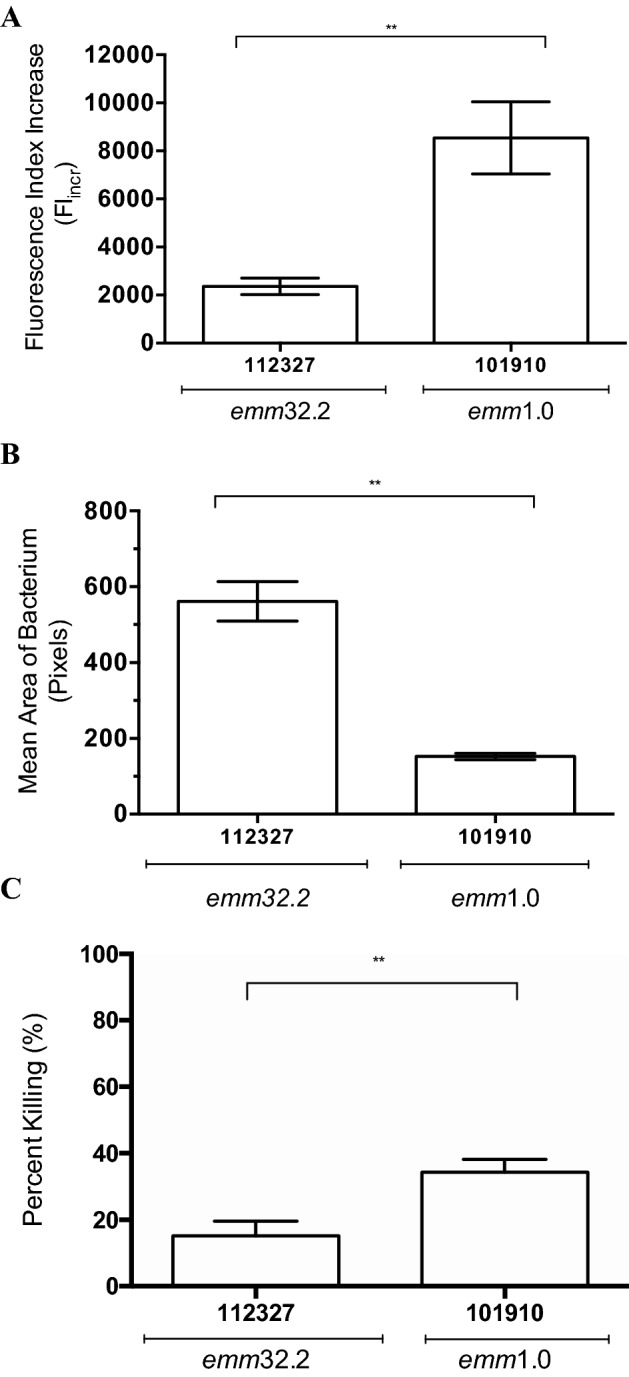
Comparison of capsule thickness, complement deposition, and OPK survival between *emm* type 32.2 (isolate 112,327) and *emm* type 1.0 (isolate 101,910). (**A**) Complement deposition (Fi). (**B**) Capsule thickness (mean-pixels) and (**C**) percent killing by macrophages. Analysed using a two-tailed Mann Whitney U-test, **p < 0.01, ***p < 0.005.

**Figure 2:**
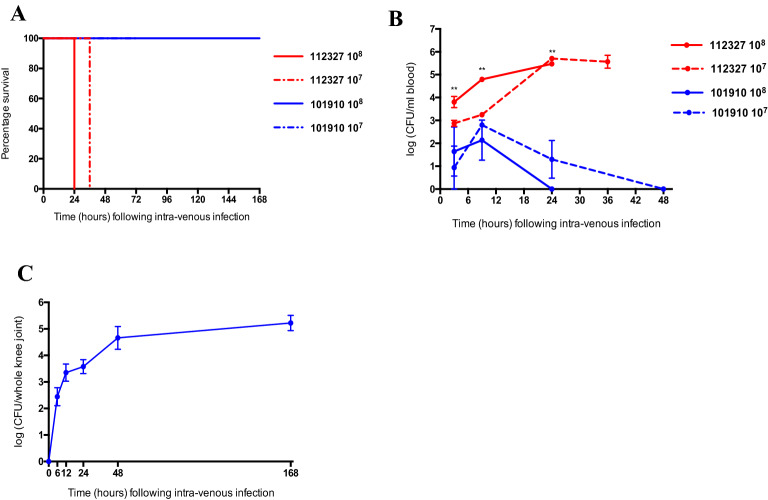
In vivo characterisation of *emm* type 32.2 (isolate 112,327) and *emm* type 1.0 (isolate 101,910) in a model of invasive GAS infection. (**A**) Kaplan Meier plots representing percentage survival of CD1 mice (n = 10 per group) following 10^8^ and 10^7^ CFU intravenous infection with *emm* type 1.0 (isolate 101,910) and *emm* type 32.2 (isolate 112,327). (**B**) The bacterial CFU in blood for each isolate and infectious dose over time. (**C**) The bacterial CFU in knee joints of CD1 mice (n = 10, knee joints n = 20) following intravenous infection with 10^7^ CFU (50 μl) of *emm* type 1.0 (isolate 101,910). **p value < 0.01 when analysed using a one-way ANOVA followed by a Kruskall-Wallis multiple comparisons test.

An invasive GAS model was used to compare the virulence of *emm* type 1.0 (isolate 101,910) and *emm* type 32.2 (isolate 112,327) in vivo*.* Both isolates were from an invasive clinical phenotype but had significantly distinct phenotypic differences, with the e*mm* type 32.2 isolate 112,327 exhibiting significantly lower complement deposition (Fig. 1A), a thicker capsule (Fig. 1B), and increased resistance to opsonophagocytic killing in comparison to the *emm* type 1.0 isolate 101,910 (Fig. 1C). An invasive GAS model was used to compare the virulence of *emm* type 1.0 and *emm* type 32.2 isolates in vivo*.* Following intravenous infection with 10^8^ colony-forming unit (CFU) of isolate *emm* type 32.2, 100% of mice scored ++ lethargy and were euthanised by 24 h post-infection, with a tenfold lower dose of infection (10^7^ CFU), all mice showed signs of lethargy by 24 h and by 36 h post-infection scored ++ lethargy (Fig. 2A). In contrast, none of the mice infected with either 10^8^ or 10^7^ CFU of *emm* type 1.0 showed any signs of bacteraemia and all survived (Fig. 2A). In time point experiments, mice infected with 10^8^ CFU of *emm* type 32.2 had significantly higher bacterial loads in their blood at all time points post infection compared to those infected with *emm* type 1.0. This was also the case by 24 h for *emm* type 32.2 infected at tenfold lower dose (10^7^ CFU), suggesting that *emm* type 32.2 is significantly better adapted to survival and proliferation in blood.

There was no detectable CFU of *emm* type 1.0 in blood by 24 h (at 10^8^ dose) and by 48 h (at 10^7^ dose), demonstrating a clear difference in blood survival (Fig. 2B) suggesting that *emm* type 1.0 was either less well adapted to survive in blood or was able to rapidly translocate out of blood and into tissue. Mice infected with *emm* type 1.0 began to show symptoms of joint deformities by 24 h, which progressed until the end of the experiment. Bacteria were recovered from the knee joints at a mean log 2.4 CFU/knee joint as early as 6 h (Fig. 2C), and the bacterial load continued to increase up to a mean log 5.2 CFU/knee joint by the end of the experiment (day 7) (Fig. 2C).

### Comparison of SLO production and activity in emm type 32.2 and 1.0 isolates

**Figure 3:**
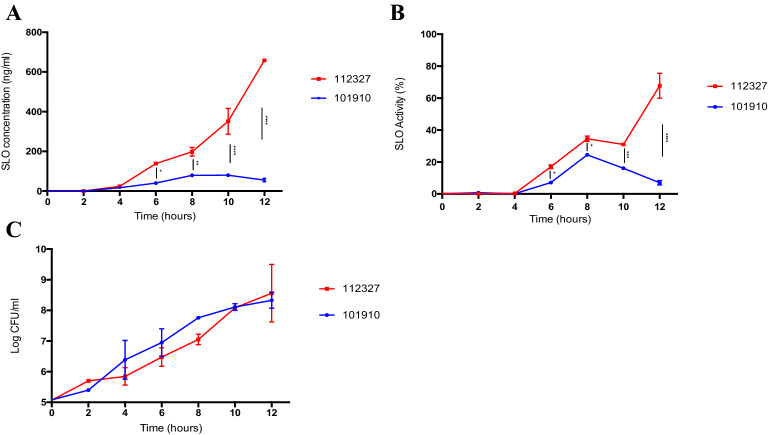
Comparison of streptolysin production and activity in *emm* type 32.2 and *emm* type 1.0. (**A**) Concentration of streptolysin (ng/ml) secreted into the supernatant by *emm* type 32.2 (isolate 112,327) and *emm* type 1.0 (isolate 101,910) over time, measured by a custom made SLO-ELISA. (**B**) SLO haemolytic activity and (**C**) growth kinetics of isolates displayed as CFUs. *p value < 0.05, **p value < 0.01, ***p value < 0.005, and ****p value < 0.001 when analysed using a two-way ANOVA followed by a Bonferroni’s multiple comparisons correction.

To explain the differences in overall mouse survival, bacterial virulence and proliferation in blood between the two *emm* type 32.2 and 1.0 isolates, we quantified the amount of SLO secreted into the supernatant by each isolate in vitro (at equivalent CFU). The quantity of SLO directly secreted into the supernatant by the bacteria during growth phase in planktonic culture was recorded. The concentration (ng/ml) of SLO produced by *emm* type 32.2 increased rapidly over time compared with *emm* type 1.0. *Emm* type 32.2 produced significantly more SLO from 6 h onwards until the final time point at 12 h (p = 0.015 to  < 0.0001). *Emm* type 1.0 produced a small amount of SLO initially but the concentration did not continue to increase beyond 8 h (Fig. 3A).

The haemolytic activity of SLO secreted by *emm* type 32.2 and *emm* type 1.0 isolates followed the same pattern as that of the amount of toxin secreted. *Emm* type 32.2 SLO was significantly more haemolytic from 6 h until the final time point at 12 h compared to *emm* type 1.0 SLO (p = 0.028 to  < 0.0001) (Fig. 3B). Hence, *emm* type 32.2 secreted significantly more SLO than *emm* type 1.0 and demonstrated significantly more haemolytic toxin at equivalent CFU. We found no significant difference in bacterial growth for both isolates across all time points, with almost identical CFU loads at 10 and 12 h (Fig. 3C). Further, at time points 10 and 12 h the difference in SLO concentration and activity was greatest, suggesting that bacterial growth rate and CFU load were not responsible for observed SLO differences between isolates.

### In vivo recovered *emm* type 1.0 has reduced production and activity of SLO

**Figure 4:**
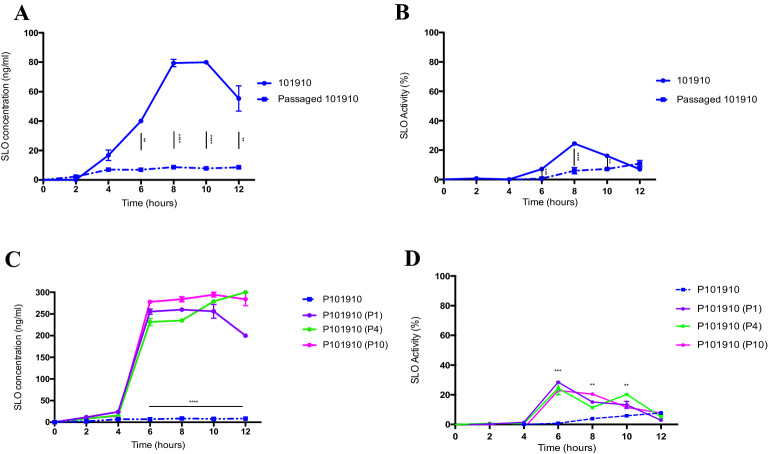
Comparison of streptolysin production and activity in emm type 1.0 grown in vitro or recovered from in vivo. (**A**) Concentration of streptolysin (ng/ml) secreted into the supernatant by *emm* type 1.0 (isolate 101,910) grown in vitro or recovered from knee joints (P101910) and then grown in vitro. (**B**) SLO haemolytic activity. (**C**) After subsequent in vitro passaging of in vivo recovered P101910 in Todd-Hewitt broth, the concentration of streptolysin (ng/ml) was measured, (**D**) and the SLO haemolytic activity. **p value < 0.01, ***p < 0.005 and ****p value < 0.001 two-way ANOVA followed by a Bonferroni’s multiple comparisons correction.

The *emm type* 1.0 translocated from blood to knee joints during course of infection whereby no CFUs were recovered from blood by 24 h, but were recovered from knee joints at equivalent timepoint. Bacteria recovered from the knee joints were quantified for SLO secretion. We found that the in vivo recovered *emm* type 1.0 secreted significantly less SLO into the supernatant over the 12 h in vitro growth phase. There was significantly less SLO secreted from 6 h onward to that originally produced by *emm* type 1.0 grown in vitro at equivalent CFU (p = 0.009 to  < 0.0001) (Fig. 4A). The haemolytic activity of in vivo tissue recovered bacterial SLO was also significantly lower from 6 to 10 h at equivalent CFU (p = 0.0007 to  < 0.0001) (Fig. 4B).

SLO production and activity of in vivo tissue recovered *emm* type 1.0 was also assessed when grown multiple times in vitro to see if SLO production and activity levels were fixed or adapted to growth conditions. After the first growth phase in THYG medium, the concentration of SLO reverted to a high SLO production phenotype (p =  < 0.0001) (Fig. 4C). Haemolytic activity also significantly increased from 6 to 10 h (p =  < 0.005) (Fig. 4D), suggesting that factors in vivo caused *emm* type 1.0 to suppress its SLO production rather than any fixed phenotype. Intravenous infection with in vivo recovered *emm* type 1.0 (10^7^ CFU), resulted in higher bacterial numbers and greater proliferation in the knee joint earlier in infection (Figure S1A).

### Concentration and activity of secreted SLO have significant impact on virulence in vivo

**Figure 5:**
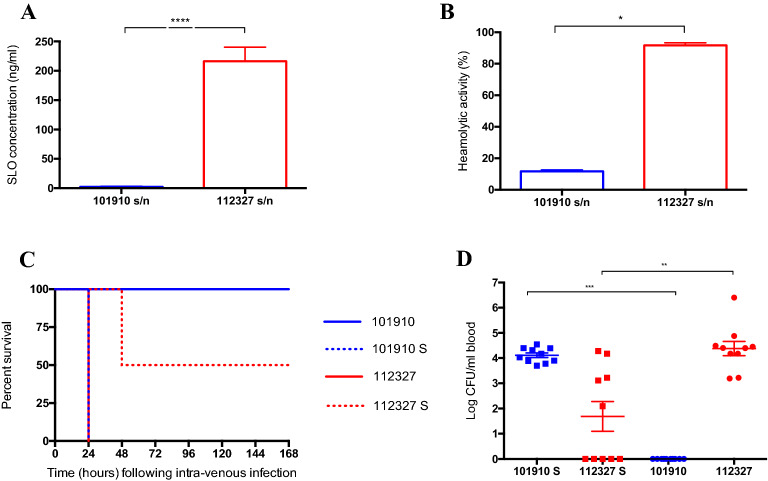
Effect of concentration and activity of secreted SLO on virulence in vivo. (**A**) Concentration of streptolysin (ng/ml) and (**B**) haemolytic activity in infection doses of *emm* type 1.0 (isolate 101,910) and *emm* type 32.2 (isolate 112,327), when prepared in 1 ml of PBS, incubated at room temperature for 30 min. (**C**) Kaplan Meier survival plots representing survival of CD1 mice (n = 10 per group) when intravenously infected (10^8^ CFU) with isolates 101,910 and 112,327, and 112,327 bacteria re-suspended in supernatant from 101,910 challenge dose (112,327 S) or 101,910 bacteria re-suspended in supernatant from 112,327 challenge dose (101,910 S). (**D**) Bacterial burden in blood 24 h after infection with isolates 101,910 and 112,327 and swapped supernatant isolates as above. **p value < 0.01, ***p < 0.005 and ****p value < 0.001 when analysed using a one-way ANOVA followed by a Kruskall-Wallis multiple comparisons test.

To investigate the effect of secreted SLO on virulence in vivo, we quantified the amount and activity of SLO released into the challenge inoculum (prior to infection of mice) of *emm* type 1.0 and *emm* type 32.2. In a challenge inoculum of 10^8^ per 50 μl, *emm* type 32.2 showed significantly higher SLO concentrations (p = 0.012) (Fig. 5A) and haemolytic activity (p = 0.01) (Fig. 5B) than *emm* type 1.0. This had a direct effect on survival in vivo, where all mice infected with *emm* type 32.2 died from the infection, while those infected with *emm* type 1.0 all survived (Fig. 5C).

To further investigate the effect of secreted SLO on infection dose and survival, the supernatants between *emm* type 32.2 and *emm* type 1.0 were swapped prior to infection of the mice. Infection doses were prepared in 1 ml of PBS, incubated at room temperature for 30 min, immediately prior to infection bacteria were pelleted by centrifugation and the supernatants of the two challenge doses were swapped. Mice were infected with either *emm* type 32.2 bacteria re-suspended in supernatant from *emm* type 1.0 challenge dose or *emm* type 1.0 bacteria re-suspended in supernatant from *emm* type 32.2 challenge dose. In contrast to original challenge dose infections, the supernatant swap infected mice exhibited the opposite phenotype. The normally non-lethal *emm* type 1.0, now resulted in 100% mice reaching the predetermined endpoint (++lethargy) when infected with supernatant from *emm* type 32.2, and the normally lethal *emm* type 32.2 isolate became less virulent, leading to only 50% death as compared to 100% death previously (Fig. 5C).

Moreover, we determined the bacterial load in blood 24 h post-infection. As previously observed, there were no CFUs of *emm* type 1.0 in blood at 24 h, but a significant 4 Log increase in CFUs was observed when *emm* type 1.0 was infected with the supernatant swap dose, clearly suggesting that the high concentration of SLO present in *emm* type 32.2 supernatant was enabling proliferation and retention of *emm* type 1.0 in blood as compared to its normal condition of being cleared from blood (Fig. 5D). In contrast, *emm* type 32.2 challenge dose with *emm* type 1.0 supernatant infected mice had significantly lower CFUs in blood at 24 h in comparison to when infected with its original supernatant (p = 0.0079) (Fig. 5D).

### SLO deficiency significantly reduces bacterial load and increases in vivo survival

**Figure 6:**
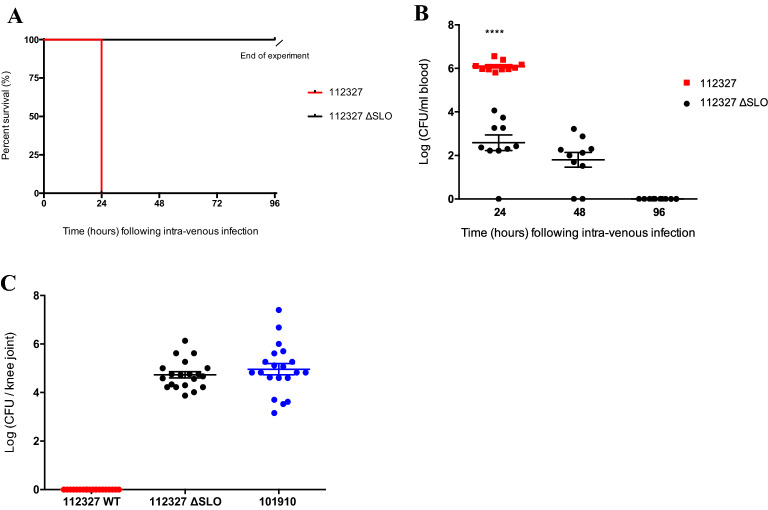
SLO deficiency increases in vivo survival and switches phenotype. (**A**) Kaplan Meier plots representing percentage survival of CD1 mice (n = 10 per group) following 10^8^ CFU intravenous infection with isolates *emm* type 32.2 (isolate 112,327) and *emm* type 32.2 (isolate ΔSLO 112,327). (**B**) The bacterial CFU in blood for each isolate over time. (**C**) Bacterial load in knee joints (n = 20) at 24 h. ****p value < 0.0001 when analysed using a two tailed Mann–Whitney U test.

To further assess the involvement of SLO in the virulence of *emm* type 32.2 in vivo*,* we generated an isogenic SLO deletion mutant (*emm* type 32.2 112,327 ΔSLO mutant) in which the SLO gene was deleted and replaced by a spectinomycin resistance gene through allelic exchange. As previously observed with *emm* type 1.0 (Fig. 2A), all mice infected intravenously with the *emm* type 32.2 ΔSLO mutant survived till the end of the experiment (96 h post infection), compared to mice infected with the wildtype parent, whom all succumbed to infection by 24 h post-infection (Fig. 6A). The bacterial load in blood was 3.5 log lower in the *emm* type 32.2 ΔSLO mutant by 24 h post infection compared with the mice infected with the wild type isolate (p =  < 0.0001) (Fig. 6B). The bacterial burden of the *emm* type 32.2 ΔSLO mutant decreased over time until 96 h post infection when the bacteria were completely cleared from the blood (Fig. 6B). These results indicate that in the absence of the toxin, bacteria were less able to establish an infection in the blood or were able to translocate elsewhere. By 24 h of infection with *emm* type 32.2 ΔSLO the mice presented with joint deformities comparable to infection with *emm* type 1.0. In addition to joint deformity, high bacterial load of *emm* type 32.2 ΔSLO was confirmed in the knee joints compared with the wild type parent (Fig. 6C). We detected no difference in the bacterial burden in the joints between *emm* type 32.2 ΔSLO mutant and *emm* type 1.0 (Fig. 6C). The kinetics of the infection was the same across the two different isolates and suggesting that SLO is the key factor determining virulence in vivo in these strains.

**Figure 7:**
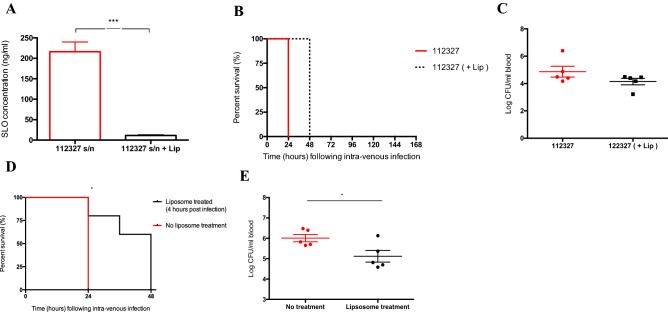
Liposome SLO treatment reduces bacterial burden and increases survival. (**A**) Concentration of streptolysin (ng/ml) in infection doses of *emm* type 32.2 (isolate 112,327) and *emm* type 32.2 (isolate 112,327) after liposome treatment. (**B**) Kaplan Meier survival plots representing survival of CD1 mice (n = 5) when intravenously infected (10^8^ CFU) with *emm* type 32.2 (isolate 112,327) and after treatment of *emm* type 32.2 (isolate 112327) supernatant with 4 μg/ml of liposomes. (**C**) Bacterial burden in blood 24 h after infection. (**D**) Kaplan Meier survival plots comparison representing survival of CD1 mice (n = 5 per group) when intravenously infected (10^8^ CFU) *emm* type 32.2 isolate 112,327 and after injection of liposomal mixture 4 h after infection. (**E**) Bacterial burden in blood 24 h after infection. Survival data was analysed using the Log-rank (Mantel-Cox) test (*p < 0.05). Data displayed as mean SEM and analysed using a two tailed Mann–Whitney U-test (*p < 0.05, ***p < 0.005).

In addition, mice infected with wild type *emm* type 32.2 and treated with liposomes (known to sequester cholesterol dependent cytolysins both in vitro and in vivo, showed a reduction of the bacterial burden in blood and an attenuation of invasive GAS infection, leading to an increased survival (Fig. 7)^31,32^.

## Discussion

In this study we show that levels of SLO production and activity determine two distinct in vivo phenotypes; *emm* type 32.2 isolates which produced SLO in high levels and with high activity and *emm* type 1.0 isolates, which were the exact opposite with low levels of SLO production and of low activity. This correlated directly with their different in vivo phenotypes i.e. high virulence in bacteraemia models accompanied by short host survival (*emm* type 32.2) and low virulence in septic arthritis models accompanied by long term host survival (*emm* type 1.0). Also, further in vitro experiments were carried out to characterise the pathogenic phenotype of *emm* type 32.2 in comparison with e*mm* type 1.0. Experiments that assessed the bacteria’s ability to resist killing by phagocytosis, capsule thickness and complement were performed on the isolates.

We found that the level and subsequent activity of SLO at time of initial infection, determined the disease phenotype, with high levels of SLO driving invasive disease and low levels sustaining joint infections. When removing SLO from the in vivo environment, either by gene deletion or by significantly reducing SLO (by supernatant swap or liposome sequestration), we were able to demonstrate a complete reversal in the in vivo phenotypes of these *emm* isolates.

SLO is a major virulence factor for GAS, expressed by nearly all strains, and with amino acid sequence homology highly conserved between strains^33^. Multiple roles in pathogenicity in vivo have been attributed to SLO, and studies have shown that SLO is important in the evasion of the host response via a number of mechanisms. Timmer et al., demonstrated that GAS induced rapid macrophage and neutrophil apoptosis due to the effects of SLO^24^, and further work in the field has demonstrated that SLO impairs neutrophil oxidative burst, preventing the bactericidal action of neutrophils^34^. The effects of the general presence of secreted SLO in the blood stream has been less well studied, SLO has been implicated in driving inflammation including the well documented evidence on activation of the NLRP3 inflammasome^35^. Therefore it seems likely that SLO production and activity is important for GAS in invasive bacteraemia infections, yet there have been no studies to date to show that SLO itself could be driving disease phenotype*.* Studies have shown that there are differences in the expression of the SLO gene which regulates the production of secreted SLO^36^, and that specific invasive variants can be isolated post in vivo passage^13^. Recently the role of CovR/CovS operon has been implicated in driving expression levels of SLO through mutations in promoter regions^17^. In our isolates we found that there were no amino acid substitutions or SNPs in CovR, CovS, HasA, HasB, HasC, HylA or Nga (NADase) which may have led to phenotype heterogeneity between the *emm 32*.2 and *emm* 1.0 *strains*^30^. In addition to this, it has been shown that in vivo conditions can result in differential expression of certain proteins; a study looking at exotoxins SpeA and SpeB found that in vivo host and/or environmental signals induced SpeA gene expression while suppressing SpeB expression, phenotypes which could not be induced under in vitro conditions^15^.

This study demonstrates that SLO level and activity determines invasiveness or chronicity during infection. The role of SLO was further investigated using supernatant switching, an SLO-deficient mutant and SLO sequestration by cholesterol rich liposomes. When the supernatant of *emm* type 32.2 was replaced with *emm* type 1.0 supernatant, the amount of SLO in the challenge inoculum was significantly reduced and 50% of the mice challenged were able to clear the infection, a delayed invasive phenotype was observed with mortality at 48 h instead of 24 h with *emm* type 32.2 and its original supernatant. This demonstrated that without the initial high SLO concentration in the challenge inoculum there is an attenuation of virulence. The bacteria may secrete SLO during the infection but the initial challenge concentration remains the key determinant. Interestingly, when we reversed this experiment and used the supernatant from the challenge inoculum of high SLO secreting *emm* type 32.2 and co-infected that with *emm* type 1.0, we saw a complete change in the clinical phenotype, whereby *emm* type 1.0 was now able to successfully proliferate in the blood resulting in host death. Taking both of these results together, they indicate that the amount of SLO that is initially secreted is key to virulence in the early stages of infection, and it is possible for the host to successfully clear the bacteria when SLO concentrations are low.

To consider how the complete removal of SLO affects the progression of invasive infection, an SLO-deficient mutant was used. Our results demonstrate that mice infected with this SLO mutant had a significantly higher rate of survival than mice infected with its parent wild type strain. Surprisingly, we found that the SLO deficient mutant sequestered in the knee joints causing septic arthritis as previously seen during infection with the low SLO secreting *emm* type 1.0. The results clearly show that GAS strains lacking SLO and or low SLO producing GAS strains are severely impaired in their ability to cause bacteraemia and that lack (or reduced levels) of SLO enables the bacteria to proliferate within host joints. There have been a number of previous studies using SLO mutants which have found that virulence is attenuated, although the relative importance of SLO would appear to be dependent on disease model used^23,28,29,37^. For example, Limbago et al., used a subcutaneous invasive skin infection model to study the virulence of SLO-deficient mutants, where they found that although there were increased survival times of mice infected with SLO deficient strains, the absence of SLO itself did not limit dissemination from the wound into the vasculature^28^. In contrast to this, a later study by Sierig et al., found that during a skin infection model initiated by intraperitoneal infection there were no changes to survival using an SLO deficient mutant^23^. A more recent study looking at the emergence of an invasive *emm* type 89.0 clade, showed that elevated SLO producers are significantly more virulent than low SLO producers^25^. Based on our findings here, we speculate that the low production of SLO (or SLO deficiency) prevents the ability of GAS to cause bacteraemia while enhancing its capability to translocate into the joints. Low SLO secreting isolate *emm* type 1.0 which effectively colonises the joints, adapts further to the joint niche by selecting for low secreting SLO variants. This may be a selection pressure applied from environmental signals in the joint as when the isolate is recovered from the joints and placed under growth conditions in vitro it reverts to producing significantly more SLO (Fig. 4C). It is able to produce more SLO than previous suggesting that GAS is highly sensitive to environmental signals and can change its phenotype rapidly. Moreover, deletion of SLO in *emm* type 32.2 resulted in a complete reversal of in vivo phenotype. Different strains of bacteria that commonly infect the joint including GAS and others such as *S. aureus* have varying degrees of tropism to the joint, thought to be due to differences in adherence characteristics and toxin production^38^. Further work should be done to examine other specific bacterial characteristics alongside the up or down regulation of SLO that may be involved in the ability of different GAS isolates to preferentially induce septic arthritis or sepsis.

The results presented here have important implications for our understanding of GAS pathogenesis. We conclude that levels and activity of SLO is key to determining whether GAS infection follows a highly invasive and virulent pattern leading to host death or whether it follows a chronic pattern of long term joint infection. The fact that these disease phenotypes are not fixed is highly interesting, as it suggests that GAS is sensitive to environmental signals and can change its phenotype rapidly. Indeed, by artificially affecting SLO levels, we have shown that one disease phenotype can easily be switched into another. This has significant implications for therapy and vaccines^39^ as anti-SLO based treatments may not be the complete answer to protection against all forms of GAS infection.

## Materials and methods

### Epidemiological study design and collection of isolates

As previously published in Cornick et al., between January 2010 to September 2012, the Respiratory and Vaccine Preventable Bacteria Reference Unit (RVPBRU) in the United Kingdom confirmed a total of 14 cases of *emm* type 32.2 invasive GAS in the Merseyside area. Over the same time period, 30 non-*emm* type 32.2 invasive GAS infections were collected alongside 20 non-invasive pharyngitis GAS isolates supplied by the Royal Liverpool University Hospitals Trust and Alder Hey Children’s Hospital^30^. This study used a representative from the *emm* 32.2 isolates (isolate 112327) and an invasive *emm* 1.0 isolate collected during the outbreak timeframe (isolate 101910). All isolates were stored in Microbank™ beads prior to study.

### Bacterial culture conditions

Isolates were grown on blood agar base (Oxoid) supplemented with 5% fresh horse blood and incubated overnight at 37 °C in a candle jar. Liquid cultures were prepared in Todd Hewitt broth with 0.5% yeast extract and 0.5% glucose (THYG) and grown overnight at 37 °C. Stocks of GAS in exponential growth phase were prepared by inoculating THYG broth with overnight cultures (1:40), and incubating at 37 °C for 3–4 h. Glycerol was added (20% v/v) and stocks were stored at − 80 °C.

### Measurement of capsular thickness

Capsule thickness was measured using the FITC-dextran zone of exclusion method, as previously described, with minor modifications^40^. Exponential phase cultures were centrifuged at 3000 g for 10 min, and the pellet re-suspended in PBS. 10 µl of bacterial suspension was mixed with 1 µl of 2000 kDa FITC-dextran (Sigma-Aldrich) and pipetted onto a microscope slide. The Nikon Eclipse 80i fluorescence microscope (100 × magnification) was used to view the slides and photographs were taken using a Hamamatsu C4742-95 camera. ImageJ was used to determine the zone of exclusion (area in pixels), a value proportional to capsular thickness.

### Complement deposition assay

The complement deposition assay was based on a previously published method^41^. Briefly, bacteria was added to brain heart infusion (BHI) broth, incubated at 37 °C for 15 min, and centrifuged. The supernatant was removed and the pellet was washed and re-suspended for incubation in PBS with 20% human serum (pooled from five individuals) and 1% gelatin veronal buffer. After washing, the pellets were re-suspended in mouse-anti-human-C3 in PBS (Abcam) and incubated at 37 °C for 30 min. Washing was repeated, and the contents were re-suspended in anti-Mouse IgG2a-APC in PBS (EBioscience) and incubated at 4 °C for 30 min in the absence of light. After washing, the remaining bacteria were re-suspended in PBS and incubated with thiazole orange (BD Cell Viability kit). Samples were acquired using the Accuri C6 flow cytometer (BD).

### Opsonophagocytosis killing assay

The ability of isolates to resist killing by macrophages was measured using an adapted protocol of a previously described opsonophagocytosis killing assay (OPKA)^42^. J774.2 macrophage cell line (ECACC) was maintained, as per standard protocols^43^. Bacteria (1 × 10^5^ CFU/ml) were opsonised with IVIg (1:4) in Hanks' Balanced Salt Solution (HBSS) (plus Ca2+/Mg2+, 5% fetal bovine serum) for 20 min at 37 °C with shaking at 180 rpm. Next, 1 × 10^5^ J774.2 cells were incubated with 5 × 10^2^ CFU of opsonised bacteria and 10 µl of baby rabbit serum complement (37 °C, 45 min, 180 rpm). The CFU count in each well was then determined. Percentage killing was calculated from CFU remaining compared to control samples without J774.2 cells.

### In vivo models of invasive GAS infection

Seven-week-old CD1 mice (Charles River) were intravenously injected with PBS containing either 10^7^ or 10^8^ CFU of GAS in exponential growth phase. Following infection, mice were monitored for physical signs of disease using a standard scoring system^44^. CFU counts were performed on blood collected at time points by tail bleeding. Mice were humanely culled when they were scored “++lethargic” and blood tissue was collected for CFU counts. In the septic arthritis model 50 mice were intravenously infected with 10^7^ CFU of *emm* type 1.0 isolate 101,910. Five mice were culled immediately after infection, and 10 mice were culled at each of the following time points post-infection 6, 12, 24, 48, and 168 h. Knee joints were recovered and CFUs enumerated. To make passaged stocks two CD1 mice were infected IV with 10^7^ bacteria. The mice were monitored to ensure that 24 h following infection they were at least a score of 1 on the arthritic index, a scoring system, which evaluates the intensity of arthritis, based on macroscopic inspection. The mice were humanely culled, the knee joints collected and bacteria were recovered to make bacterial stocks.

In vivo experimental procedures were approved by the University of Liverpool Ethical and Animal Welfare Committee and carried out under the authority of the UK Home Office Animals Scientific Procedures Act 1986 (UK Home Office Project Licence number P86DE83DA). Experiments were conducted and reported in accordance with the ARRIVE guidelines (Animal Research: Reporting of in vivo experiments).

### Arthritic index calculation

Briefly limbs were inspected visually at regular intervals (6, 12, 24, 48, 72 h post injection). Arthritis was defined as visible erythema and/or joint swelling of at least one joint. To evaluate the intensity of arthritis, a clinical scoring (arthritic index) was carried out by using a system where macroscopic inspection yielded a score of 0 to 3 points for each limb (1 point = mild swelling and/or erythema; 2 points = moderate swelling and erythema; 3 points = marked swelling and erythema and occasionally ankylosis). The arthritic index was constructed by dividing the total score by the number of animals used in each experiment group.

### Streptolysin ELISA design and method

Samples were thawed at room temperature. Plates (R&D systems) were coated with 1 μg/well monoclonal SLO antibody (Abcam) in PBS (Peprotech) at 4 °C overnight. Plates were washed at each step with Peprotech washing buffer. After blocking (Peprotech), samples were added to the wells and incubated for 2 h at room temperature. The plate was washed (× 5) and incubated with rabbit IgG polyclonal anti-SLO antibody (Abcam) for 2 h. Anti-rabbit IgG alkaline phosphatase conjugate secondary antibody (Abcam) was diluted to 1:5000 in blocking buffer, and after washing, was added and incubated for 30 min. After washing, alkaline phosphatase yellow liquid substrate (PNPP) (Abcam) was added and incubated for 30 min in the dark, to stop the reaction 1 M Sodium Hydroxide (NaOH) was used. The plate was loaded on to a Multiskan Spectrum (Thermo) and the absorbance measured at 405 nm. All ELISAs were carried out with control wells which had all reagents added except samples or diluted SLO. Duplicate samples of each time point was measured on a single plate and repeated independently. Each plate contained six two-fold dilutions of a known concentration of SLO. The results were analysed using Sigma Plot and a standard curve developed to generate concentrations in ng/ml.

### Haemolytic activity assay

The haemolytic activity of SLO secreted in culture supernatant was measured as previously described, with minor modifications^45^. Bacteria-free supernatants were incubated at room temperature for 10 min with 20 mmol/l of dithiothreitol (Sigma-Aldrich). Supernatant was aliquoted into two tubes; 25 µg of water-soluble cholesterol (inhibitor for SLO activity) was added to one. Both tubes were incubated at 37 °C for 30 min, followed by the addition of 2% sheep erythrocytes/PBS suspension to each sample and further incubation at 37 °C for 30 min. PBS was added to each tube the samples were centrifuged at 3000 × g for 5 min. Each sample was transferred to a 96-well plate and the OD_541nm_ was measured.

### Generation of slo detion GAS mutant

An isogenic SLO knockout mutant of strain *emm* type 32.2 isolate 112,327 was constructed through double-crossover allelic replacement of SLO with aad9 (encoding spectinomycin resistance). Regions directly upstream and downstream of SLO (~ 1000 bp each) were amplified by PCR using primers SLO112327-up-F and SLO112327-up-R, SLO112327-down-F and SLO112327-down-R respectively (Table S1), which introduced BamHI restriction sites into the PCR products. These fragments were stitched together in a second round of PCR using primers SLO112327-up-F and SLO112327-down-R (Table S1), generating a 2 kb fragment with a central BamHI site, which was then ligated into pGEM-T vector (Promega), generating pGEM-T-*∆slo*-2 kb. The plasmid was transformed into *E. coli* DH5α competent cells (ThermoFischer Scientific). The aad9 gene was amplified by PCR using primers aad9-F and aad9-R (Table S1). The PCR product was subcloned into pGEM-T-*∆slo*-2 kb at the BamHI restriction site, generating pGEM-T-*∆slo::aad9*, which interrupted the *slo* gene, providing a means of positive selection of transformants. The generated plasmid was transformed into *emm* type 32.2 isolate 112,327 by electroporation as previously described^46^. Transformants were recovered on THY agar supplemented with spectinomycin (100 µg/mL) at 37 °C in a candle jar for up to 72 h. SLO deletion was identified by PCR and the PCR products were sequenced to confirm authenticity of the deletion.

### In vivo invasive model- switching supernatant of isolates

Frozen bacterial stocks were thawed at room temperature and 10^7^ bacteria were prepared in 1 ml PBS. After 30 min both strains were centrifuged at 14,000 × g for 2 min, the supernatant from *emm* type 32.2 (isolate 112,327) was used to re-suspend *emm* type 1.0 (isolate 101,910) bacteria and the supernatant from *emm* type 1.0 (isolate 101,910) was used to re-suspend *emm* type 32.2 (isolate 112,327) bacteria. Mice were immediately infected. The supernatants were analysed using the SLO-ELISA to measure the amount SLO present in ng/ml. Mice were humanely culled when they were scored ‘++lethargic’ and blood tissue was collected for CFU enumeration.

### Liposomes

Liposomes were generated with cholesterol and ﻿sphingomyelin from egg yolk from Sigma and dissolved in chloroform at 100 and 50 mg/ml respectively. Lipids were mixed together with ﻿cholesterol at 66 mol/% proportion and then evaporated with nitrogen gas for 30 min. For Cholesterol: Sphingomyelin (Ch:Sm) large and small liposomes, the hydration was made by addition of PBS (ThermoFisher scientific) and incubated at 55 °C for 30 min with vortexing. To obtain small unilamellar particles, the liposome preparation was then subsequently sonicated for 30 min at 4 °C. To eliminate carboxyfluorescein, the preparation was diluted in PBS and applied to a Sephadex G-25 column in PD-10 (GE Healthcare). Particle concentration and size distribution of the liposomes generated were evaluated using the NanoSight NS300 instrument (Malvern, UK) and using Nanoparticle Tracking Analysis (NTA) software.

### Data analysis

Statistical analysis was carried out using the GraphPad Prism® version 5 statistical package (GraphPad Software, Inc. http://www.graphpad.com). The statistical significance according to the p values were summarised as follows: *p value < 0.05, **p value < 0.01, ***p value < 0.005 and ****p value < 0.001.

## Supplementary Information

Supplementary Information.
