# Supplementary material for: Streptolysin O concentration and activity is central to in vivo phenotype and disease outcome in Group A Streptococcus infection
Source: Sci Rep. 2021 Sep 24;11:19011. doi: 10.1038/s41598-021-97866-4 (PMC8463576; doi:10.1038/s41598-021-97866-4)
Supplement: Supplementary file 1 — Supplementary Information. [file 41598_2021_97866_MOESM1_ESM.docx]

**Supplementary data**

**A.**

**B.**

**Supplementary Figure 1- Comparison of knee joint CFU and arthritic index after intravenous infection with *emm* type 1.0 isolate 101910 and *in vivo* recovered *emm* type 1.0 isolate 101910.** A) The bacterial CFU in knee joints of CD1 mice (n = 10, knee joints n = 20) and B) arthritic index score following 10^7^ CFU in 50 μl intravenous infection with *emm* type 1.0 isolate 101910 (black solid line) and *in vivo* recovered *emm* type 1.0 isolate 101910 (red dashed line). ***p-value < 0.005 when analysed using a one-way Anova and Kruskal-Wallis multiple comparisons test, displayed as mean ± SEM.

**

**Supplementary Figure 2 - Growth kinetics of emm1.0 10910 and animal passaged isolates displayed as CFUs.**

**Supplementary Table 1- Primers used in single knockout of SLO in GAS.** Primer sequences in bold correspond to BamHI restriction site.

| **Name** | **Sequence 5’>3’** |
| --- | --- |
| SLO112327-up-F | GCTAGCGGAGATACTCCTGGAGC |
| SLO112327-up-R | CTGA**GGATCC**CATGTCCTTCATACCTTTTTATC |
| SLO112327-down-F | CATG**GGATCC**TCAGGACTGGTTCAAGAG |
| SLO112327-down-R | GCGCGAGACACACTGGTCCTGAC |
| aad9-F | GAACTAGT**GGATCC**CCCGTTTG |
| aad9-R | CAATACGGGATAATACCGCGC |
